# Python as a Federation Tool for GENESIS 3.0

**DOI:** 10.1371/journal.pone.0029018

**Published:** 2012-01-20

**Authors:** Hugo Cornelis, Armando L. Rodriguez, Allan D. Coop, James M. Bower

**Affiliations:** 1 Cornelis H. Research Imaging Institute, University of Texas Health Science Center at San Antonio, San Antonio, Texas, United States of America; 2 Rodriguez A. L. Research Imaging Institute, University of Texas Health Science Center at San Antonio, San Antonio, Texas, United States of America; 3 Coop A. D. Department of Epidemiology and Biostatistics, University of Texas Health Science Center at San Antonio, San Antonio, Texas, United States of America; 4 Bower J. M. Research Imaging Institute, University of Texas Health Science Center at San Antonio, San Antonio, Texas, United States of America; University of Alberta, Canada

## Abstract

The GENESIS simulation platform was one of the first broad-scale modeling systems in computational biology to encourage modelers to develop and share model features and components. Supported by a large developer community, it participated in innovative simulator technologies such as benchmarking, parallelization, and declarative model specification and was the first neural simulator to define bindings for the Python scripting language. An important feature of the latest version of GENESIS is that it decomposes into self-contained software components complying with the Computational Biology Initiative federated software architecture. This architecture allows separate scripting bindings to be defined for different necessary components of the simulator, e.g., the mathematical solvers and graphical user interface. Python is a scripting language that provides rich sets of freely available open source libraries. With clean dynamic object-oriented designs, they produce highly readable code and are widely employed in specialized areas of software component integration. We employ a simplified wrapper and interface generator to examine an application programming interface and make it available to a given scripting language. This allows independent software components to be ‘glued’ together and connected to external libraries and applications from user-defined Python or Perl scripts. We illustrate our approach with three examples of Python scripting. (1) Generate and run a simple single-compartment model neuron connected to a stand-alone mathematical solver. (2) Interface a mathematical solver with GENESIS 3.0 to explore a neuron morphology from either an interactive command-line or graphical user interface. (3) Apply scripting bindings to connect the GENESIS 3.0 simulator to external graphical libraries and an open source three dimensional content creation suite that supports visualization of models based on electron microscopy and their conversion to computational models. Employed in this way, the stand-alone software components of the GENESIS 3.0 simulator provide a framework for progressive federated software development in computational neuroscience.

## Introduction

The GEneral NEural SImulation System (GENESIS, http://genesis-sim.org/) is a general purpose simulation platform originally developed to support the simulation of neural systems ranging from sub-cellular components and biochemical reactions to complex models of single neurons, simulations of large networks, and systems-level models.

GENESIS was one of the first broad scale modeling systems in computational biology to encourage modelers to develop and share model features and components. For these people, it was the object-oriented approach taken by the simulator along with its high-level simulation language and Script Language Interpreter (SLI), that allowed the exchange, modification, and reuse of models or model components. It was this community of developers and users that ultimately drove the development of the GENESIS platform.

The development of the GENESIS simulator was initiated during the 1980's through research projects that addressed specific scientific questions in computational neuroscience. Simulator functionality was expanded through life cycles of research project extension. For example, libraries for kinetic pathway modeling were added for projects investigating how signaling networks store learned behavior [1] and how light controls photoreception by regulating calcium release from intracellular calcium stores [2]. A fast implicit solver was developed for complex Purkinje cell modeling [3], [4] and more recently synaptic learning rules have been implemented [5]. In principle such linear or single-threaded development processes can continue forever. However, repetitive extension of GENESIS with source code of diverse functions and origin has made the code structure so complicated that it is almost impossible to extend. The unitary nature and density of the source code has ultimately created a‘monolithic’ application. This marginalizes user contributions to simulator functionality, updates and releases have become less frequent, and the software life cycle is moved from extension to maintenance.

GENESIS 3.0 (G-3) is a major reconfiguration and update of the GENESIS simulation system. This reconfiguration is based on the *Neurospaces Project* (http://neurospaces.sourceforge.net/) which was initiated in 1998 as a development center for software components for computational neuroscience simulators. It embodies many software components, each of which has been developed in full isolation. In it, the core GENESIS simulator functionality has been restructured such that it is the first neural simulator to comply with a more modular design, the Computational Biology Initiative federated software architecture (referred to as the CBI architecture, described in Materials and Methods and in more detail in [6]). The CBI architecture is specifically designed to support the integration of stand-alone software components and applications by using common integration technologies such as modern scripting languages. This not only results in improved simulator performance, portability, and code reusability but also enables both the use of new script parsers and user interfaces and the capacity to communicate with other modeling programs and environments and external model and data analysis and presentation software.

In the following sections we illustrate the use of the general purpose Python scripting language for making high performance simulation software coded in system programming languages accessible to neuroscientists and biologists. We note that equivalent functionality is available from G-3 through the Perl (http://www.perl.org/) scripting language.

## Materials and Methods

In the following sections we overview the GENESIS software platform and its recent reconfiguration to comply with the CBI architecture (described below). We also introduce two scripting languages employed by G-3 (Python and Perl) and a simplified wrapper and interface generator (SWIG). We outline how they are suitable as federation tools for the continued extension of GENESIS functionality. Our approach to software development is based on, but not limited to, such languages and provides a paradigm that simplifies the extension, modification, and customization of complex neurobiological simulation software, not only for developers but also most importantly for users.

Starting from an existing source code base, and learning from previous experience in simulator development, G-3 is a modularization of the core functions of the GENESIS simulator. The guiding principles that define the implementation and function of the G-3 simulator are based on the CBI architecture, a modular abstracted architecture that layers the data in a simulator and separates data representations from the algorithms that process them.

### GENESIS 1 and 2

GENESIS simulations are constructed from model components that receive inputs, perform calculations on them, and then generate outputs. Model neurons are constructed from basic parts, such as segments, and variable conductance ion channels. (Note: ‘Segment’ is a high level term employed to describe different parts of the biological model of a dendritic morphology. The equivalent low level (computational) term is ‘compartment’. It refers to the numerical representation of a segment.) Channels are linked to their segments which in turn are linked to form multi-segment neurons of any desired level of complexity. Neurons may be then be connected to form neural circuits [7].

The GENESIS SLI was a high-level simulation language that provided a framework within which a modeler could extend the capabilities of the simulator and manipulate models or model components by exchange, modification, and reuse. The SLI interpreted statements in the GENESIS simulation language and constituted the operating system ‘shell’. User-defined SLI scripts were then used to glue together the pieces of a simulation. These scripts also controlled the graphical objects used to define the front end of a simulation and the GENESIS data handlers. It was the commands the SLI recognized and the many GENESIS ‘objects’ available for constructing models and simulations that have most powerfully assisted in the sharing of model features amongst the broader modeling community.

### Scripting Languages

Historically, there have been fundamental differences between the Unix shells and system programming languages such as C or C++ and scripting languages such as Perl [8], Python [9], Rexx [10], Tcl [11]. System programming languages typically start from the most primitive computer elements, usually the ‘words’ of memory. They are designed to manage the complexity of building data structures and algorithms from scratch and generally require pre-declared data types. Alternatively, as a replacement for shell scripts and shell communication pipes, scripting languages assume the existence of a set of software components and are primarily intended to assemble or glue together these components. In this way, scripting languages operate at a higher level than system programming languages in the sense that on average a single statement in a scripting language does more work. For example, a typical statement in a system programming language executes about five machine instructions, whereas, in a scripting language a typical statement may execute hundreds or thousands of machine instructions [12].

The strongly typed nature of system programming languages discourages reuse. Scripting languages, on the other hand, have actually stimulated significant software reuse. They use a model where interesting components are built in a system programming language and then glued together into applications. This division of labor provides a natural framework for reusability. When well-defined interfaces exist between components and scripts, software reuse becomes easy. In this sense scripting and system programming are symbiotic. Used together, they produce programming environments of exceptional power where applications can be developed five to ten times more rapidly than when a system programming language alone is used.

**Figure 1:**
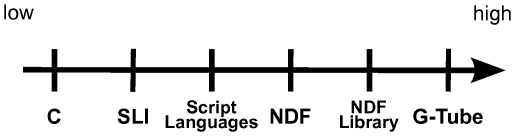
Levels of programing in GENESIS 3.0. The concept hierarchy used for communication by GENESIS developers. C Coding: System programing languages are used to program individual simulator components and extend simulator functionality. SLI: Indicates the level of the GENESIS-2 Script Language Interface in the concept hierarchy. Script Languages: Python and Perl are used to interface individual simulator components. NDF: The native declarative file format used to build models and model components. NDF Libraries: Model components such as membrane channels and synapses provide a library of functional components available for accelerated model development. G-Tube: The GENESIS graphical user interface allows users to access models and model components for the rapid development of single cell and network models.

Figure 1 gives the levels of programing available in G-3. They range from the C coding employed to create the independent components, through Python and Perl interface scripting, the declarative NDF file format used for model construction and the associated reusable NDF libraries coding membrane channels and synapses, to the highest level of coding via the G-Tube GUI. Also indicated is the location of the SLI in this coding hierarchy.

In summary, system programming languages are well suited for building functional software components where there is a requirement for computing speed because data structures and algorithms are complex, whereas, scripting languages are well suited for assembling applications where the complexity is in the connections. With an increasing requirement for software integration, scripting is providing an important programming paradigm.

### Python

Python is a powerful dynamic programming language comparable to Perl, Ruby, or Scheme. In September 2011, 4% of internet references to programing and scripting languages were to Python, making it the 8th most popular programming language (see Tiobe Index at http://www.tiobe.com/index.php/content/company/Home.html. Note: Ratings are based on the number of skilled engineers world-wide, courses, and third party vendors. The search engines Google, Bing, Yahoo!, Wikipedia, YouTube and Baidu are used to calculate the ratings). It combines considerable power with very clear syntax and has modules, classes, exceptions, high level data types, and dynamic and loose typing. It runs on many hardware architectures, integrates with scientific and user interface libraries, and new modules are easily written in C or C++ (or other languages, depending on the chosen implementation). It is also usable as an extension language for applications written in other languages that need easy-to-use scripting or automated interfaces. It is currently the highest ranked scripting language.

### Perl

Perl was one of the first open source scripting languages. First released in 1987 (http://groups.google.com/group/comp.sources.unix/msg/bb3ee125385ae25f?pli=1), it is unique in that it is very much informed by linguistic principles. Originally developed as a scripting language for UNIX, it aimed to blend the ease of use of the UNIX shell with the power and flexibility of a system programming language like C. With over 20 years of development and nearly half a million lines of code, Perl now runs on over 100 different platforms (http://www.perl.org/about.html). Currently, there are over 18,000 open source modules available from the Comprehensive Perl Archive Network (CPAN, http://www.cpan.org/), assisting in system integration, scientific application, and user interface development. Via the CPAN *Inline* module, Perl integrates seamlessly with both system programming languages such as C and C++, and scripting languages including Python. (Note: *italicized* text indicates the names of Python modules, directory paths, and file names, whereas, Typewriter text is reserved for Python coding examples and the dimensions of physical quantities. **Bold** text indicates the names of software components in G-3.) Perl supports object-oriented programming, functional programming, and procedural programming paradigms. In September 2011, nearly 2.5% of internet references to programing and scripting languages were to Perl, making it the 9th most popular programming language (see Tiobe Index at http://www.tiobe.com/index.php/content/company/Home.html).

### Meta-Programming in Perl and Python

Meta-programming is a programming technique where a program generates a new program and then executes it. This technique is employed by the G-3 Python and Perl bindings to generate an additional layer of script code that provides increased flexibility for defining models and simulations. A predefined Python or Perl data structure defines high-level interfaces that are translated into strings containing Python or Perl code such as class and method definitions. During program initialization these are bound to the run-time environment using the Perl or Python *eval* functions.

### SWIG for Federated Software Integration

SWIG is a software development tool that connects programs written in system programming languages such as C and C++ with high-level scripting languages such as Python or Perl. SWIG was chosen to facilitate the use of these bindings in G-3. It controls most aspects of wrapper generation and automates the generation of the required Perl and Python interfaces. SWIG uses a layered approach to build extension modules where different parts are defined in either C or the chosen scripting language. The C layer contains low-level wrappers whereas the script code is used to define high-level features. Considerably more flexibility is obtained by generating code in both languages as an extension module can then be enhanced with support code in either language. Table 1 gives an overview of the resulting code. As expected, low-level software components emphasize low-level languages and contain more code (e.g. C), whereas, high-level software components emphasize high-level languages and contain less code (e.g. Python, Perl).

**Table 1:** Comparison of Hand-Written and Generated Code (in Byte Counts).

Language:	C (H)	C (G)	Perl (H)	Perl (G)	Python (H)	Python (G)
**Model Container**	1,832,580	4,416,163	30,406	207,638	14,568	250,178
**Heccer**	1,163,991	1,575,615	57,565	107,261	1,586	171,219
**NS-SLI**	1,448,636	483,641	4,603	2,802	—	—
**SSP**	829	2,323	55,063	—	—	—
**Studio**	—	—	174,923	—	—	—
**G-Shell**	—	—	28,142	—	623	836

A comparison of hand-written (H) and automatically generated (G) code that supports the functionality of the independent components of the GENESIS simulation platform. Software hand-written in a system programming language such as C contains more code (in bytes) than the equivalent functionality replicated in a scripting language. The amount of automatically generated code is considerably greater than that written by hand.

### The CBI Federated Software Architecture

The CBI architecture provides a modular paradigm that places stand-alone software components into logical relationships. Each software module is an independent component that allows development and maintenance to be implemented concurrently. In this section we summarize the data-flow related concepts of the CBI architecture. For an in-depth explanation see the accompanying paper [6].

**Figure 2:**
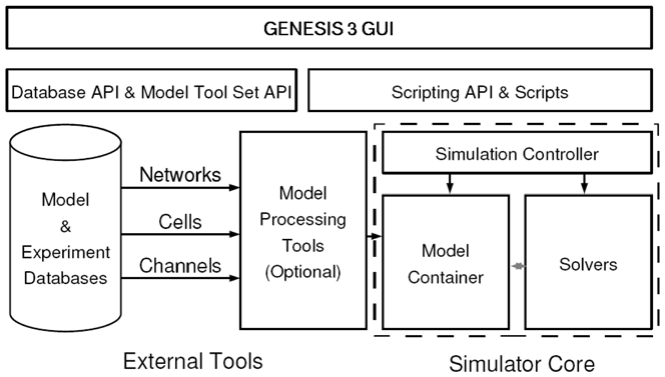
Relation of components in the Computational Biology Initiative (CBI) federated software architecture. The CBI architecture defines the relationships between the necessary components of a computer-based neural simulation system. The architecture contains three layers that include a graphical user interface (GUI) connected to the functional components of a simulator by an interposed layer of application programming interfaces (APIs) and scripts. Components in the lowest layer are interconnected by APIs while their particular functionality is needed. GENESIS 3 is the first simulator to be implemented in compliance with the CBI architecture.

The important data-flow related components of the architecture are shown in Figure 2. On the bottom left are databases of neuronal models or experimental data that can be accessed by the simulator. Optional model processors (e.g. the Reconstruct interface) load a model into the **Model Container** which stores a model in memory and makes it available to other software components in different formats. One function of the **Model Container** is to translate biological concepts and properties into mathematical concepts that can be understood by the mathematical solvers. Thus, importantly and unlike other existing neural simulators, the mathematical solvers are independent of the biological representation of a model. A simulation controller orchestrates the actions taken by the **Model Container** (e.g. when to load a model, the definition of the stimulus, and when to export a model) and mathematical solvers (e.g. when to fetch a model from the **Model Container**, when to start calculations, and what the output variables are).

A scripting layer allows the simulation system to be driven from multiple scripting languages. Python and Perl are currently supported, as is (for backward compatibility) the GENESIS SLI. The G-3 GUI is shown at the top of Figure 2 and is being developed entirely in Python. It supports many functions and allows models to be imported from databases or constructed from scratch, the exploration of model structure and parameters, and the visualization of variables and model behavior.

Within the CBI paradigm each software component is self contained and can be run independently. This facilitates the interoperability of software obtained from different sources and has several important advantages for software development, including: (1) Reduced complexity of software components compared to a unitary system, (2) simplified documentation of components in terms of inputs and outputs, (3) simplified development and testing of components as stand alone software, (4) clear delineation of scope for the development of new components, and (5) independent update, enhancement, or replacement of individual components when needed, making the life cycle of a modular architecture smoother than that of a non-scalable application.

The CBI architecture provides a framework for the integration of independent software components into a functioning simulator using a scripting language of choice. Here, we specifically illustrate the use of Python for this purpose.

### G-3 as a CBI Compliant Simulator

G-3 is a major revision and update of the GENESIS system. The core simulator functionality is restructured, with a more modern modular software design referred to as the CBI architecture. This not only results in improved simulator performance and portability, but allows the use of alternate script parsers and user interfaces that provide the ability to communicate with other modeling programs and environments.

Much existing software such as GUI libraries and plotting libraries, are application neutral. Other software packages are tailored to the needs of computational neuroscience. The Neurospaces project (http://www.neurospaces.org/) provides core software components for the G-3 simulator [13]. These include, the (1) **Model Container**: Stores two representations of a model, the first is conceptual and can be regarded as an enumeration of biological concepts and their relationships, the second is an expanded mathematical representation that, if complete, can be simulated, (2) **Heccer**: A fast compartmental solver based on the GENESIS *hsolve* object that can be instantiated from C, Python, Perl or other scripting languages, (3) **SSP** (Simple Scheduler in Perl): Binds **Heccer** and the **Model Container**, and activates them correctly, such that they work together on a single simulation, (4) **Studio** and **G-Tube**: Contain graphical tools for model construction, exploration, and simulation, (5) **G-Shell** (G-3 Interactive Shell): Dynamically loads other software components in an interactive environment, and the (6) **Project Browser**: For inspection of projects and simulation results. For completeness, we also mention (7) **NS-SLI**: The G-3 component that provides backward compatibility for the GENESIS SLI. All software can be downloaded from the GENESIS web site (http://genesis-sim.org/download/) and extensive installation instructions with examples are available from the GENESIS documentation website (http://www.genesis-sim.org/userdocs/genesis-installation/genesis-installation.html). Simulator correctness can be established by running automated regression and integration tests.

## Results

PyGENESIS (http://www.cs.caltech.edu/~mvanier/hacking/pygenesis/pygenesis.tar.gz) was a version of GENESIS developed in the late 1990's. It replaced the standard GENESIS SLI with a Python interface. This Python-enabled version of GENESIS was never publicly released. However, with the increased sophistication of the Python platform and reconfiguration of GENESIS to comply with the CBI architecture, Python interfaces have been developed for several of G-3's core simulator components. While it is possible to drive each component in isolation from these interfaces, here we show how individual components may be integrated via Python to create a simple simulator, explore a dendritic morphology, and connect to external applications that support sophisticated model visualization and morphological analysis.

### A Python Enabled Neural Simulator

Python uses modules to group related functions. G-3 employs Python's module system to group functions that provide an interface to each of its software components. As an example, the G-3 Python module *nmc* contains functions to simplify the storage of neuron models in computer memory. This module is a simple front-end to the **Model Container**, a G-3 component which for efficiency is coded in the system programming language C. Likewise, *Heccer* is a wrapper module for the **Heccer** component which in turn is an interface to a low-level single neuron solver written in C. Python bindings for the **Discrete Event System** to facilitate network modeling also exist.

Here we show a simple high-level Python script that runs a simulation of a single cylindrical segment. It is defined by standard values for the parameters of membrane resistance (RM), axial resistance (RA), and membrane capacitance (CM). (Note: This script is written for clarity of presentation rather than compactness or efficiency. Python version 2.6.6 is used on Linux Ubuntu 10.10 Maveric.) These parameters are given by their specific values (in SI units) as commonly reported in the literature, instead of their actual values scaled to the compartment surface area as used by a mathematical solver [14]. The script defines a Python function *run_simulation* that will load and run a model when invoked from a system command line on an appropriately configured computer. The script can also be imported into G-3 as a Python module, thus allowing access to this function. For convenience, we call this Python module *example*.

″″″

Comment: Python script running a simple model with G-3.

″″″

from g3.nmc import ModelContainer

def RunSimulation (simulationTime):

 timeStep = 1e-5

#------------------------------------------------------------------------------

# Create a model container with a neuron cell and a dendritic segment

#------------------------------------------------------------------------------

 my_nmc = ModelContainer()

 my_cell = my_nmc.CreateCell (“/cell”)

 my_segment = my_nmc.CreateSegment (“/cell/soma”)

 my_segment.SetParameters(

  {

  “Vm_init”: −0.0680,

  “RM”: 1.000,

  “RA”: 2.50,

  “CM”: 0.0164,

  “ELEAK”: −0.0800,

  “DIA”: 2e-05,

  “LENGTH”: 4.47e-05,

  }

  )

# Apply current injection to the soma

 my_segment.SetParameter(“INJECT”, 1e-9)

#------------------------------------------------------------------------------

# Create a Heccer for computing the neuron model stored by the model container.

#------------------------------------------------------------------------------

 from g3.heccer import Heccer

 my_heccer = Heccer(name="/cell”, model = my_nmc)

 my_heccer.CompileAll()

#-----------------------------------------------------------------------------

# Create an output object.

#------------------------------------------------------------------------------

 from g3.experiment.output import Output

 my_output = Output(“/tmp/output”)

#-----------------------------------------------------------------------------

# Link the output object to the address of the computed variable of interest.

#------------------------------------------------------------------------------

 my_output.AddOutput(“output”, my_heccer.GetAddress(“/cell/soma”, “Vm”))

#-----------------------------------------------------------------------------

# Create an array of the objects that participate in the simulation.

#------------------------------------------------------------------------------

 schedulees = []

 # schedule heccer

 schedulees.append(my_heccer)

 schedulees.append(my_output)

#-----------------------------------------------------------------------------

# Advance all the particpating objects for the duration of the simulation.

#------------------------------------------------------------------------------

 currentTime = 0.0

 while currentTime<simulationTime:

  currentTime+ = timeStep

  for schedulee in schedulees:

   schedulee.Advance(currentTime)

 my_heccer.Finish()

 my_output.Finish()

#------------------------------------------------------------------------------

# Main program executes a simulation of 0.5 seconds.

# The if statement allows use of this file as an executable or as a library.

#------------------------------------------------------------------------------

if _--_name_--_ =  = ‘_--_main_--_’:

 RunSimulation(0.5)

### Contrasting Levels of Expressibility

The CBI architecture allows G-3 to accommodate many user interfaces. As an example, the compartmental solver **Heccer** can be driven stand-alone from C code, Python, or Perl to run the simplest models, or it can be interfaced with the **Model Container** to run more realistic multi-compartment models based on morphological data. To illustrate this flexibility we now compare the above Python script with alternative implementations in C and the GENESIS SLI.

In the C code there is an abundance of low level detail that interfaces directly to the solver. For example, compartments are identified by their position in an array and parameters such as RM and CM must be provided as an unlabelled ordered sequence of their actual values (scaled to the compartment surface area).

The complexity of the GENESIS SLI interface falls between that of the Python interface and the C code interface. (Note: the G-2 SLI is supported by G-3 through its backward compatibility component **NS-SLI**.) While compartments and parameters have names and numerical values are given in a format used by solvers.

#### C Code Implementation

#include “heccer/compartment.h”

struct Compartment compSoma = 

{

 // type of structure

 { MATH_TYPE_Compartment, },

 −1, // no parent compartment

 4.57537e-11, // Cm

−0.08, // Em

−0.068, // InitVm

0, // Inject

360502, // Ra

3.58441e+08, // Rm

};

// compartment and channel mapping

int piC2m[] = { 0, −1, };

// model definition

struct Intermediary inter = 

{ 1, &compSoma, NULL, piC2m, };

// include commands for simulation

#include “main.c”

#### GENESIS SLI Implementation

create neutral/cell

create compartment/cell/soma

setfield/cell/soma dia 2e-05

setfield/cell/soma len 4.47e-05

setfield/cell/soma Cm 4.57537e-11

setfield/cell/soma Em −0.0800

setfield/cell/soma Vm_init −0.068

setfield/cell/soma inject 1e-9

setfield/cell/soma Ra 360502

setfield/cell/soma Rm 3.58441e+08

reset step 0.5 -time

While script language bindings are suitable for construction of toy models from scratch, it is better to use a domain specific language to construct the various parts of a model. For example, the **Model Container** is installed with a library of domain specific model components where the standard Hodgkin-Huxley channels are provided in the file *channels/hodgkin-huxley.ndf*. These channels can be included in the Python *example* given above by adding the statements:

 my_segment.ImportChild(“channels/hodgkin-huxley.ndf::/k”)

 my_segment.ImportChild(“channels/hodgkin-huxley.ndf::/na”)

The **Model Container** can export models constructed in a scripting language as a library for incorporation into new models or for use with other G-3 components such as the **Project Browser**. These new models can then be imported by a call to the **Model Container** *Read* method. For example, importing a Purkinje cell model with over 4,000 compartments may be done with the following statement:

my_nmc.Read(“cells/purkinje/edsjb1994.ndf”)

After importation, the **Model Container** provides a set of functions to analyze the structure of a model morphology. For example, the names of the most distal segment of each dendrite can be obtained with:

my_nmc.Query(“segmentertips/Purkinje”)

### Interactive Query and Simulation

The **G-Shell** integrates other G-3 components and makes their functions available through an interactive environment available from a command line. Coded in Perl, the **G-Shell** is a communication abstraction layer for the **Model Container**, **Heccer**, **SSP**, **DES** and the **Studio**. After the **G-Shell** has been started from a system shell with

genesis-g3

the list of loaded software components can be printed to the screen with the command:

list components

Each loaded software component will be shown with associated status information that helps diagnose possible problems. For example, after correct initialization of the **Model Container** the status information appears as:

model-container:

 description: internal storage for neuronal models

 integrator: Neurospaces::Integrators::Commands

 module: Neurospaces

 status: loaded

 type:

  description: intermediary

  layer: 2

Integration of the **G-Shell** with the **Model Container** allows for real-time analysis of the quantitative and structural aspects of a neuronal morphology. The library of model components that is installed with the **Model Container** provides a definition of a model Purkinje cell in the file *cells/purkinje/edsjb1994.ndf*. The command:

ndf_load cells/purkinje/edsjb1994.ndf

makes this model Purkinje cell available for interactive analysis. Alternatively, if the model is encoded in a GENESIS SLI script with the name *PurkM9_model/CURRENT9.g*, the command *ndf_load* can be replaced by *sli_load*:

sli_load PurkM9_model/CURRENT9.g

This command imports the model that is specified in the SLI script without running the simulation. A similar command (*pynn_load*) is currently in active development to interface with the PyNN network modeling environment [15].

Given the name of one of its dendritic segments, the number of branch points between the segment and the soma can be determined. After indicating with the command *morphology_summarize* which paths of the dendritic tree to examine, the parameter SOMATOPETAL_BRANCHPOINTS contains the result, which can be obtained with:

 morphology_summarize/Purkinje

 show_parameter/Purkinje/segments/b1s06[182] SOMATOPETAL_BRANCHPOINTS

If a dendritic segment contains a synaptic channel, it can be stimulated with a precomputed spike train stored in a file named, for example, *event_data/events.yml*:

set_runtime_parameter/Purkinje/segments/b1s06[182]/Purkinje_spine_0/head/par/synapse

 EVENT_FILENAME “event_data/events.yml”

 Finally, following the addition of an output for the somatic membrane potential:

 add_output/Purkinje/segments/soma Vm

A simulation can then conveniently be started using:

run/Purkinje 0.1

This simulation produces the somatic response to the given dendritic stimulus in a file named, by default, */tmp/output*.

To query the parameters of the stimulated compartment the model can then be analyzed using the graphical front-end of the **Studio** with the command:

explore

**Figure 3:**
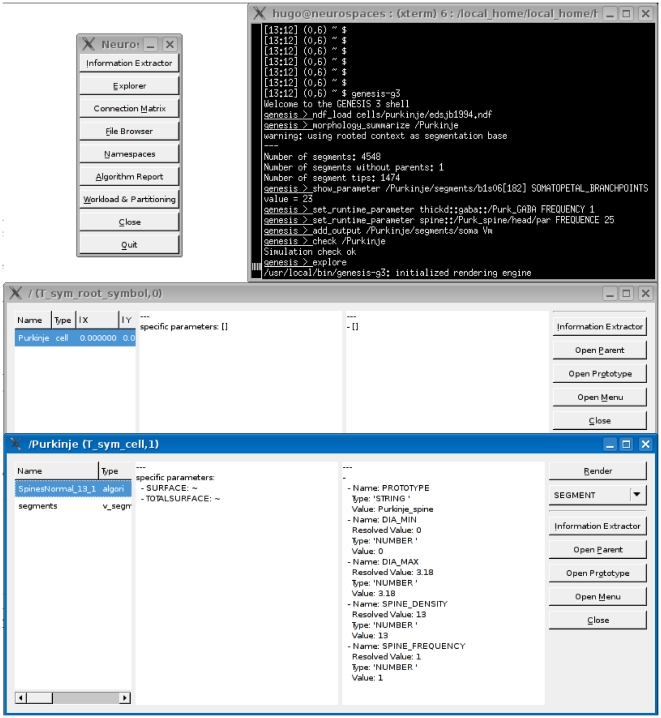
Querying a model in GENESIS 3.0. The **Studio** is a component of the reconfigured GENESIS software platform. It can be used to query the parameters of individual compartments in a multi-compartment model neuron. The **Studio** also renders 3D morphology of dendrites and generates overviews of network models.

Figure 3 shows sample output of running this command. Other capabilities of the **Studio** include rendering morphologies in three dimensions and generating overviews of network models (not shown). In the next section we explore some of the more graphical capabilities of G-3.

### Integration of Pre-existing Applications and Libraries

The GENESIS Graphical User Interface (GUI) was supported by the X-Window System Output and Display Utility for Simulations (XODUS). XODUS provided graphical objects that could be connected to model components from within the SLI. Rather than providing a full GUI instance, the flexibility of XODUS came from its infrastructure which allowed modelers to easily develop new GUIs dedicated to their particular research or teaching projects. (Note: The official GENESIS software distribution contained both simple and sophisticated example GUIs.) However, the XODUS paradigm inevitably resulted in modelers contaminating their model script with GUI related statements.

As mentioned above, one advantage of the CBI architecture is that it defines how to interface simulator components with external applications. An obvious example is the use of existing 3D graphics software to examine and edit the spatial properties of a model neuron morphology. Others include, integration with external graphing and windowing software to plot the values of solved variables against simulation time, or to allow the construction of button-rich tutorial applications.

GUI libraries typically communicate with other software components using an event based system. The functional core of such a system is an event dispatching loop, usually called the *main loop*. The binding between a button click event and the *main loop*, and the visual layout of most contemporary GUI applications is conveniently constructed using one of a number of freely available user interface builders. One such builder is *wxFormBuilder*. (Note: This builder is a user interface designer for the *wxPython* toolkit and the Linux desktop environment GNOME. It is available from http://wxformbuilder.org/.) It can be used to construct a GUI with visual elements such as menus and buttons and writes a description of these elements and their bindings to a file known as an XML resource (XRC) file. The GUI definitions in this file can then be rendered with the *wxWidgets* library and its Python front end *wxPython*. Further integration with additional G-3 specific data bindings ensures that, for example, the data produced by a mathematical solver flows to a widget that plots the value of a variable against time. This functionality replaces the XODUS paradigm, which required SLI scripting to connect GUI components to model components and simulation actions, with a more contemporary paradigm that separates simulator and model scripts from GUI related statements.

In the following example, we create a *wxPython* application class called *G3App*. This demonstrates the Python scripting required to connect the software components that create a small GUI for G-3. We specifically show how to initialize the application (implementation of method *OnInit*), how to run a simple simulation based on the previous *example* (method *OnRun*), and how to plot output (method *Plot*). To achieve this, it is assumed that a XRC file with the name *G3.xrc* can be found that describes a GUI with one frame (here, *mainFrame*) which allows the simulation duration to be set via a text control widget and contains a button to start the simulation.

**Figure 4:**
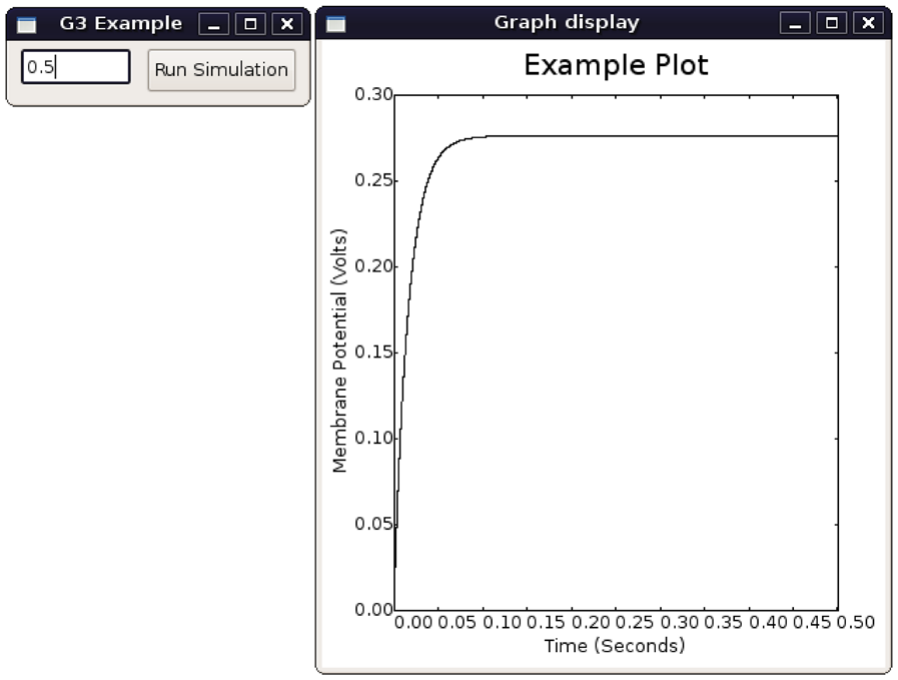
Simple Integration of G-3 with the *wxPython* widget set. The CBI architecture defines a separation between GUI statements and peripheral code. This allows GUI construction kits to be used for the creation of a user-friendly interface to a simulation. In this example *wxFormBuilder* was used to construct a text control widget and a button to start the simulation using its standard drag & drop interface. The same approach can be used for the development of research and educational projects.

The first lines of code in the script load the necessary Python modules which in turn load low-level libraries coded in a system programming language. The widget output is shown in Figure 4.

import DataPlot

import example

import wx

from wx import xrc

class G3App(wx.App):

def OnInit(self):

 # Load the XML resource file

 self.res = xrc.XmlResource(‘G3.xrc’)

 # Declare objects for each part of the gui,

 # we fetch the objects from the loaded XML resource

 # via the name we gave each component in the builder.

 self.frame = self.res.LoadFrame(None, ‘mainFrame’)

 self.runButton = xrc.XRCCTRL(self.frame, ‘runButton’)

 self.durationTextCtrl = xrc.XRCCTRL(self.frame,duration‘TextCtrl’)

 # Bind the gui objects to methods.

 #

 # Note: frame is the main component inthe MainLoop so this

 # is what we set the binding to.

 self.frame.Bind(wx.EVT_BUTTON, self.OnRun, self.runButton)

 # Display our simple gui.

 self.frame.Show()

 return True

# An action to do when the run button is pushed.

# This will run the simulation with the given time

# and plot the output.

def OnRun(self,evt):

 simulationTime = float(self.durationTextCtrl.GetValue())

 print “Simulation time is:” + str(simulationTime)

 print “running simulation…”

 example.RunSimulation(simulationTime)

 print “Simulation Complete!”

 print “Plotting output”

 self.Plot(‘/tmp/output’)

##

# Plots data output from a data file.

#

# wxPython sizer & layouts

# This is the alternative to using an

# XRC specification, this however is a small

# example.

def Plot(self,datafile):

 plotwindow = wx.Frame(self.frame, −1, “Graph display”, (480,300))

 plotpanel = wx.Panel(plotwindow, −1)

 self.dataplot = DataPlot.DataPlot(plotpanel, −1,

  ‘/tmp/output’,

  ‘Example Plot’,

  ‘Time (Seconds)’,

  ‘Membrane Potential (Volts)’)

 vbox_sizer = wx.BoxSizer(wx.VERTICAL)

 vbox_sizer.Add(self.dataplot, 1, wx.EXPAND)

 plotpanel.SetAutoLayout(True)

 plotpanel.SetSizer(vbox_sizer)

 plotpanel.Layout()

 plotwindow.Show()

# Our main function where we perform the main loop

if _--_name_--_  =  =  ‘_--_main_--_’:

 app = G3App(False)

 app.MainLoop()

In this example we have shown how the CBI architecture defines a separation between GUI statements and peripheral code such as input and output specifications, and model construction. Besides allowing common GUI construction kits to be used for the development of research and educational projects, our approach also allows G-3 to interface with highly specialized GUI kits.

### Interfacing GENESIS with External Applications

As an example of how to interface GENESIS with specialized external applications, we now show how to validate and analyze models of the morphology of small dendritic segments obtained from electron microscopy data. The required geometrical algorithms are typically available in state-of-the-art rendering applications such as Blender (http://www.blender.org/). Blender is a free open source 3D content creation suite available for all major operating systems that have Python enabled bindings (Note: One restriction is that code must be run from inside the Blender specific Python interpreter.) In our example it replaces the functionality otherwise provided by the **G-Shell**.

Over the last several years we have used electron microscopy (EM) in conjunction with Reconstruct (http://www.bu.edu/neural/Reconstruct.html, [16]) to obtain precise morphologies of small segments of Purkinje cell dendrites [17], [18].

The Reconstruct interface converts the application into a G-3 simulator component by making it CBI compliant. This allows Reconstruct data to be imported into the **Model Container**. The core of the interface implements geometrical transformation algorithms that convert EM contours provided by Reconstruct to equivalent cylinders suitable for cable modeling. The necessary conversion algorithms are accessible from the **Model Container** via Python. The geometrical properties of the cylinders are stored in the native G-3 NDF file format and algorithms provided by the **Model Container** link them to the cable parameters required by the mathematical solvers. A simulation can then be run with the *read* and *run* methods given above.

The Python interface of Blender links it to the G-3 simulator to provide, to our knowledge, the first 3D model inspection tool for EM data available directly from a neural simulator. As an example, the Python script developed above can be run from within the Blender environment. Also, via the same Python interface, simulations can be started based on the 3D image data.

**Figure 5:**
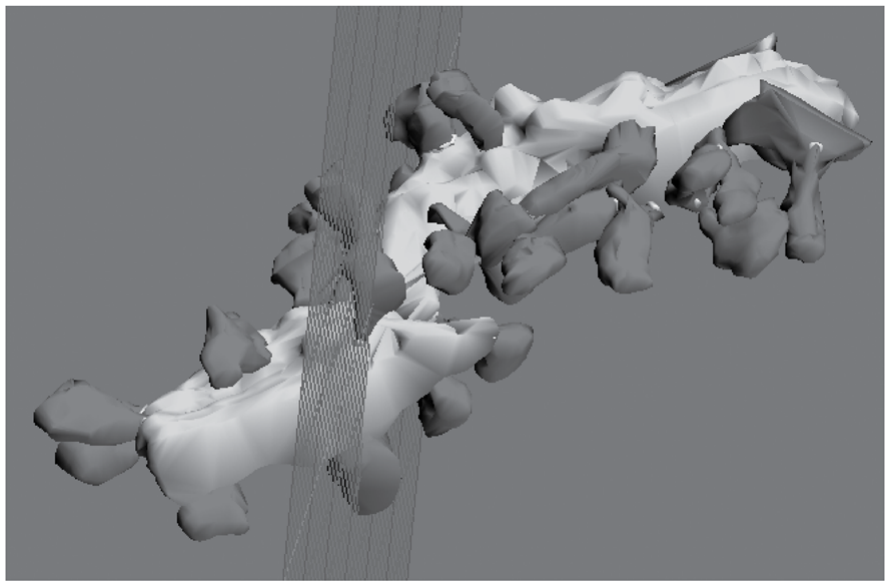
Image of reconstructed Purkinje neuron dendritic segment. Blender is an open source 3D content creation suite that can be interfaced with the GENESIS neural simulation platform to replace the functionality otherwise provided by the **G-Shell** (a stand-alone GENESIS component). The rendering functions of Blender can then be used to analyze the morphology of small dendritic segments imported from electron microscopy data.

Interactive visualization of reconstructed dendritic segments is a valuable method of model validation and is available with the integration of G-3 and Blender (see Figure 5). However, the development of small focused plugins allows for more than just this functionality. For example, 3D measurement and manipulation of neuron morphology, computation of surface areas and volumes, and the generation of 3D cross-sections and 2D cuts also becomes possible.

## Discussion

GENESIS is the first neural simulator to be reconfigured in compliance with the CBI federated software architecture. In it, the Python and Perl bindings embed similar functionality to that of the GENESIS SLI, although their purpose is different. While the SLI had as major goals the integration of model components, running simulations, and output collection, the primary goal of scripting languages has become application integration.

Through the Neurospaces project, GENESIS now provides a series of independent software components that can readily be combined to support computational modeling in the neurosciences. As examples of this scripted integration we have shown how to run a simple model using **Heccer** and the ability of the **Model Container** to query the structure of a neuronal morphology. In a third interactive example, synaptic stimulation was delivered to a Purkinje cell model.

We have described how scripting languages such as Python provide powerful integration tools and can be used to connect simulator components to general purpose external libraries and appropriately configured external applications. This expanded simulator functionality will lead to the development of considerably more sophisticated GUIs, result visualization, and data analysis.

It is notable that in the paradigm of the CBI architecture, although model parameters are stored and processed separately from stimulus protocols and the way simulations are run, the modularity of the software does not interrupt but actually enhances an integrated user experience. Simultaneously, this modularity greatly facilitates the development and maintenance of individual G-3 components.

### Extensibility in The G-3 Software Federation

An important benefit of the CBI architecture is that third party software libraries become available to users. For example, *wxFormBuilder* can be employed to generate GUI bindings for *wxPython* and integrate them with the G-3 software platform.

To demonstrate the power of our approach, we interfaced G-3 with Reconstruct and Blender. This novel integrated software platform has been used for visual inspection and validation of reconstructed dendrites by connecting a model to the geometrical and analytical tools provided by the Blender plugin library. Further, we note that it is possible to use Blender to instantiate neural simulations and, for example, collect simulation output data for movie generation.

Complementary functionality to that provided by interfacing G-3 with Blender would be available after interfacing G-3 with *neuro*Construct (http://www.neuroconstruct.org/), a software package designed to simplify the development of complex networks of biologically realistic neurons [19], [20]. Implemented in Java, *neuro*Construct uses the latest NeuroML specifications (see http://www.neuroml.org/, http://www.morphml.org/), can be used to visually validate network layout and design [21], and can be connected to Python applications (e.g. see http://www.jython.org/). In principle this allows it to be interfaced with other simulators that have Python bindings, including NEURON (http://www.neuron.yale.edu/neuron/, [22]) and NEST (http://www.nestinitiative.org/index.php/About_Us, [23]).

A serial communication framework for event delivery of action potentials to afferent synapses has been developed. Called DES, it can be integrated with the mathematical solvers of G-3 using either Python or Perl. It allows a user to stimulate one or more synapses with a specific train of afferent impulses. DES is optimized for communication over serial hardware. However, it is straight forward to extend it to support communication frameworks for parallel hardware such as those provided by the MOOSE simulator [24] and the MUSIC framework [25].

The digital reconstruction of neuronal arborization is an important step in the quantitative investigation of cellular neuroanatomy. The NeuroMorpho.Org database of neuronal morphologies (http://www.neuromorpho.org/) is a centrally curated inventory of digitally reconstructed neurons [26]. It allows extensive morphometric analysis, when linked to a simulator and provides a first step in the large scale implementation of biophysical models of electrophysiology. Interfaced with the functionality of the **Model Container** it has the capacity to accelerate the development of neuronal models by providing a direct link to data from experiments. Preliminary implementations of this functionality are now part of an automated test framework for G-3.

G-3 also significantly extends the ability of GENESIS to transparently interact with experimental technologies such as open source dynamic clamp software. As an example, the modular approach taken by the RTXI platform for dynamic clamp [27], [28] and the modular structure of G-3 mean that the solver, **Heccer**, can be directly integrated as an RTXI plug-in [29]. This greatly simplifies the required software development.

Ultimately, the extensibility of the CBI architecture provides an extremely plastic environment within which appropriately configured external applications can be integrated with a scripting language of choice.

### Implications for Neuronal Simulator Interoperability

The current generation of neural simulators can be characterized as software applications that support a user workflow extending from model construction to data analysis. Due to their simplicity [30] and ease of use [31], many of these simulators support Python bindings. They range from Monte-Carlo simulators for reaction-diffusion systems [32] and dedicated large network simulators [33] to the general purpose simulators NEURON and GENESIS [7], [34].

For these simulators, interoperability can be implemented using one of the emerging standards for model exchange such as NeuroML [35], NineML [36] or PyNN [15]. While dedicated G-3 modules supporting the use of these interoperability standards are currently under development, the G-3 platform also provides an alternative approach. By employing scripting languages a CBI compliant simulator can easily be connected to general purpose software. This provides functionality that powerfully supports the development of ‘next generation’ neural simulation platforms.

### Federated Software Development in Neuroscience

Processes of software development have traditionally been described as either cathedral-like where there is a closed development group under central direction and software releases are infrequent, or bazaar-like where software is developed by volunteers and software releases occur early and often [37], [38]. While cathederal-like software development leads to a single-threaded development cycle commonly used by commercial applications, the bazaar-like leads to multi-threaded development cycles of applications that come in different flavours. (Note: A classical example is the family of editors based on Emacs.)

Here, based on the CBI paradigm, we have outlined a solution for multi-threaded development of software components for neuroscience (for other examples of this approach to neural simulation see [39], [40]). We have given examples that use Python.

Employed in this way, the modularized design of the G-3 simulator gives rise to an ecology of software components that can be integrated in a variety of ways to provide a software development environment that is both progressive and federated.
